# PANA-Surv: A Pathway-Guided Adaptive Neighborhood Augmentation Framework Using KEGG Pathways for Multi-Omics Cancer Prognosis

**DOI:** 10.3390/genes17060597

**Published:** 2026-05-22

**Authors:** Xiaowen Cao, Yijin Zhou, Yao Dong, Xuekui Zhang, Jia-peng Mei, Jianwei Li, Yixiao Wang, Jiaming Zhuo, Hua He, Junhua Gu

**Affiliations:** 1 School of Artificial Intelligence, Hebei University of Technology, Tianjin 300401, China; 18920927551@163.com (X.C.); dongyao@scse.hebut.edu.cn (Y.D.); jiaming.zhuo@outlook.com (J.Z.); 2 Department of Mathematics and Statistics, University of Victoria, Victoria, BC V8P 5C2, Canada; xuekui@uvic.ca (X.Z.); jiapengmei@uvic.ca (J.-p.M.); 3 School of Science, Hebei University of Technology, Tianjin 300401, China; chouplu6@gmail.com (Y.Z.); 18222556898@163.com (Y.W.)

**Keywords:** multi-omics, cancer prognosis, survival analysis, KEGG pathways, graph neural network, prognostic biomarkers, bioinformatics

## Abstract

**Background/Objectives**: Integrating multi-omics data for cancer prognosis remains a challenging problem in bioinformatics because molecular profiles are high-dimensional, heterogeneous, and structured by incomplete biological relationships. Pathway databases provide biologically meaningful prior knowledge for modeling gene-level associations, but the sparsity and local incompleteness of pathway-derived networks often limit the performance of graph-based survival models. This study aimed to develop a pathway-guided framework for improving multi-omics survival prediction and identifying biologically relevant prognostic signals. **Methods**: We proposed PANA-Surv, a pathway-guided adaptive neighborhood augmentation framework for multi-omics cancer survival analysis. In this framework, KEGG pathways were used to construct gene graphs, and gene-level multi-omics profiles were encoded as node features. A conditional variational autoencoder module (PANA-VAE) was designed to enhance local representations through neighborhood reconstruction and adaptive weighting. The augmented features were then integrated into a graph convolutional survival model optimized with the Cox partial likelihood. **Results**: PANA-Surv was evaluated on 10 cancer cohorts from The Cancer Genome Atlas (TCGA). The proposed method achieved the highest mean concordance index (C-index) among all compared models and significantly outperformed Cox-EN, DeepSurv, GraphSurv, and LAGProg (all *p* < 0.01). Ablation analyses showed that both neighborhood reconstruction and adaptive weighting contributed to the observed performance gains, and KEGG-guided graph construction was more effective than alternative graph construction strategies. In a breast cancer (BRCA) case study, PANA-Surv identified 18 prognostic genes, including 12 genes supported by previous studies and 6 potentially novel candidates. **Conclusions**: These findings indicate that the integration of pathway prior knowledge with adaptive local feature enhancement can improve multi-omics survival modeling and support the identification of biologically relevant prognostic signals associated with cancer outcomes.

## 1. Introduction

Accurate prognostic assessment is an important component of precision oncology, as it supports risk stratification, outcome prediction, and the identification of molecular features associated with disease progression [1]. With the development of high-throughput sequencing technologies, multiple layers of molecular data, including gene expression, DNA methylation, and copy number variation, can now be profiled at the genome-wide scale, providing complementary views of tumor heterogeneity and disease biology [2,3,4]. These advances have created new opportunities for cancer prognosis research, but they have also introduced substantial analytical challenges because multi-omics data are high-dimensional, heterogeneous, and often difficult to integrate effectively [5,6,7]. In addition, preprocessing choices, including normalization, filtering of lowly expressed genes, batch-effect correction, and differential expression analysis, can substantially influence downstream biomarker interpretation and the reproducibility of transcriptome-based prognostic studies [8].

Pathway databases provide biologically meaningful prior knowledge for organizing gene-level molecular information. Among them, the Kyoto Encyclopedia of Genes and Genomes (KEGG) is a widely used pathway database that manually curates functional relationships among genes, gene products, biochemical reactions, and signaling processes in the form of pathway maps [9,10,11]. These pathway maps can be further represented as structured gene graphs, in which genes are treated as nodes and pathway-derived functional associations are treated as edges. By mapping multi-omics measurements onto pathway-derived graphs, it becomes possible to integrate molecular profiles with structured biological knowledge, thereby improving both model interpretability and biological relevance in prognostic analysis [12,13,14]. This pathway-guided strategy is particularly attractive for cancer bioinformatics because it allows survival models to move beyond isolated molecular features and to incorporate functional associations among genes.

Graph neural networks (GNNs) provide a natural framework for learning from such structured data and have shown promise in multi-omics integration tasks, including cancer survival prediction and subtype analysis [15,16,17]. Existing graph-based survival models typically rely on a predefined adjacency matrix derived from pathway databases or similarity networks [12,13]. However, pathway-derived graphs are often incomplete and locally sparse, and their node connectivity can vary substantially across genes. Genes with few neighbors may lack sufficient local context, whereas highly connected genes may accumulate redundant or noisy information during message passing [14]. These limitations can reduce the ability of graph-based models to learn stable and informative representations for downstream prognostic prediction.

To improve graph representation learning under imperfect graph structures, previous studies have explored feature augmentation and topology refinement strategies [16,17]. For example, conditional variational autoencoders have been used to enhance node features [18], while data-driven graph construction approaches such as k-nearest neighbor (kNN) graphs have been introduced to complement incomplete biological networks [19]. Although these strategies may improve representation quality, they can also weaken biological interpretability when the augmented structure deviates substantially from known pathway organization [20]. In pathway-guided multi-omics survival analysis, it is therefore desirable to enhance local representations while preserving biologically meaningful neighborhood relationships [21].

A recent study, LAGProg [12], moved in this direction by combining local augmentation with KEGG-guided graph modeling for multi-omics cancer prognosis. However, its neighborhood aggregation scheme assigns equal importance to all selected neighbors, which may introduce irrelevant signals in dense local regions and fail to highlight informative neighbors in sparse ones. In addition, its reliance on fixed local structure may limit its ability to adapt to heterogeneous pathway neighborhoods. These issues suggest that pathway-guided survival models may benefit from a more flexible mechanism for reconstructing and weighting local neighborhood information.

In this study, we propose PANA-Surv, a pathway-guided adaptive neighborhood augmentation framework for multi-omics cancer survival analysis. PANA-Surv uses KEGG pathways to define graph structure and encodes gene-level multi-omics profiles as node features. A conditional variational autoencoder module is introduced to reconstruct local neighborhoods and adaptively weight neighboring nodes, generating denoised augmented representations for downstream survival modeling. These features are then incorporated into a graph convolutional survival model optimized with the Cox partial likelihood. Using 10 cancer cohorts from The Cancer Genome Atlas (TCGA), we show that PANA-Surv improves prognostic performance relative to several representative survival models and supports the identification of biologically relevant prognostic genes in breast cancer.

## 2. Materials and Methods

This section describes the overview of the method, data and preprocessing, baseline methods, cross-validation setup, performance evaluation, experiment designs, and downstream analyses.

### 2.1. Overview of the Method

**Figure 1:**
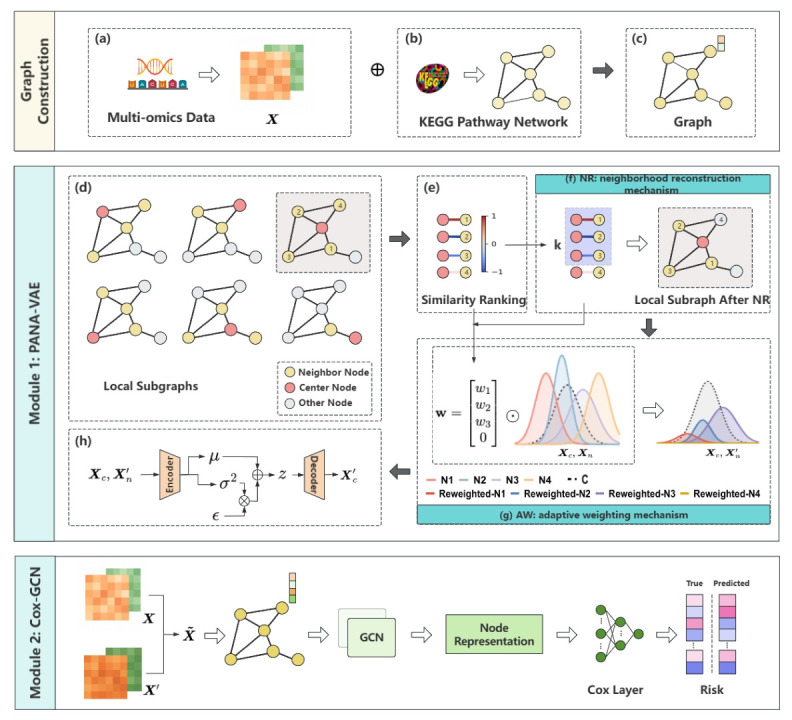
Workflow of PANA-Surv. **Graph construction** builds a pathway-guided graph by integrating multi-omics features with the KEGG pathway network (**a**–**c**). **Module 1 (PANA-VAE)** includes two mechanisms: a neighborhood reconstruction (NR) mechanism that selects the top-k most similar neighbors (**d**–**f**), and an adaptive weighting (AW) mechanism that reweights the neighborhood feature ($X_{n}$) based on computed similarities (**g**). The reweighted neighborhood features $X_{n}^{\prime }$ together with the central node features $X_{c}$ are then fed into an encoder to generate the augmented central representation $X_{c}^{\prime }$ (**h**). **Module 2 (Cox-GCN)** takes the concatenated feature matrix $\tilde{X}$ as input to a two-layer graph convolutional network with a Cox proportional hazards layer for patient risk prediction.

In this section we introduce the PANA-Surv (see Figure 1), which includes graph construction component and two modules: the PANA-VAE module, which enhances node representations through neighborhood reconstruction (NR) and adaptive weighting (AW) mechanisms, and the Cox-GCN module, which fuses the augmented features and original features to predict the survival risk.

#### 2.1.1. Graph Construction

In the graph construction stage, the pathway-guided graph (as shown in Figure 1c) can be represented as G=(V,E,X), where V denotes the set of genes (nodes) and E represents the pathway connections derived from the KEGG network (Figure 1b). The node feature matrix $X\in R^{m\times d}$ (Figure 1a) contains the multi-omics features of all genes, with m being the number of genes and d the number of omics modalities. This graph provides both the biological topology and node-level omics attributes, which serve as inputs for the subsequent PANA-VAE module.

#### 2.1.2. Module 1: PANA-VAE

##### Local Subgraphs

For each node $v_{c}$, we define its local neighborhood as $v_{n}=\{v_{n_{1}},\ldots ,v_{n_{j}}\}$, where j is the number of neighbors of node $v_{c}$. The corresponding multi-omics features are represented as $X_{c}\in R^{1\times d}$ for the central node and $X_{n}\in R^{j\times d}$ for its neighboring nodes.

Each node serves as a center to construct its local subgraph based on its directed connected neighbors (Figure 1d). In these subgraphs, the red node denotes the central node $v_{c}$*,* the yellow nodes represent its neighbors $v_{n}$, and the gray nodes indicate other genes not directly connected to $v_{c}$.

##### Similarity Ranking

For the central node $v_{c}$ and each neighbor $v_{n_{i}}\in v_{n}$, the cosine similarity is computed as $\rho_{c,n_{i}}=X_{c}\cdot X_{n_{i}}/∥X_{c}∥∥X_{n_{i}}∥$, where i=1,2,…,j. The absolute similarity values $\left|\rho_{c,n_{i}}\right|$ are then ranked in descending order (Figure 1e).

##### Neighborhood Reconstruction Mechanism

We adopt a top-k selection strategy. For each central node $v_{c}$, we retain the k=min{j,K} most similar neighbors from its original j connected neighbors, where K is a predefined hyperparameter. The reconstructed neighborhood is denoted as $V_{n}=\{v_{n_{1}},v_{n_{2}},\ldots ,v_{n_{k}}\}$. This mechanism refines the local structure of each node, pruning weak or noisy connections and focusing on biologically coherent relationships (Figure 1f).

##### Adaptive Weighting Mechanism

This mechanism assigns a normalized contribution weight to each neighbor in the reconstructed set $V_{n}$ (Figure 1g). For each selected neighbor, the weight is defined as $\omega_{i}=|\rho_{c,n_{i}}|/\sum |\rho_{c,n_{r}}|$, where $v_{n_{i}},\;v_{n_{r}}\in V_{n}$, and i=1,2,…,k. For each central node $v_{c}$, the weights of all its neighbors are collected as $\omega =(\omega_{1},\omega_{2},\ldots ,\omega_{k}{)}^{⊤}$. The weighted neighborhood features are then computed as ${X_{n}}^{\prime }=diag(\omega )X_{n}$. These two mechanisms allow each central node to adaptively form a unique local neighborhood, with neighbor weights determined by their similarities.

##### Generate the Augmented Features

As shown in Figure 1h, the central node features $X_{c}$ and the weighted neighborhood features $X_{n}^{\prime }$ are concatenated and fed into the encoder, which incorporates a latent variable z to model the conditional posterior: $q_{\phi }(z|X_{c},X_{n}^{\prime })=N\left(z;\;\mu ,\;diag(\sigma^{2})\right)$.

The encoder captures the latent dependency between each central node and its reconstructed local neighborhood through z. During training, PANA-VAE learns the joint distribution between each central node and its neighbors; the decoder then reconstructs the neighborhood features by maximizing the evidence lower bound (ELBO):

$$L=E_{q_{\phi }(z|X_{c},{X_{n}}^{\prime })}\left[\log p_{\theta }({X_{n}}^{\prime }|z,X_{c})\right]-KL\left(q_{\phi }(z|X_{c},{X_{n}}^{\prime })\|p(z)\right),$$

where p(z)=N(0,I) denotes the standard Gaussian prior.

In the generation stage, the latent variable z and the central-node feature $X_{c}$ are fed into the decoder to generate the augmented node representation: ${X_{c}}^{\prime }=\mu_{\theta }(z,X_{c}).$ Collecting all $X_{c}^{\prime }$ from all central nodes $v_{c}$ yields the global feature matrix $X^{\prime }$, which is subsequently used in the GCN-Cox module for survival prediction.

#### 2.1.3. Module 2: GCN-Cox

The concatenated feature matrix $\tilde{X}=[X,X^{\prime }]$ serves as the input to the graph convolutional network (GCN). Feature aggregation is defined as:

$$H^{\left(1\right)}=ReLU\left(\tilde{P}\tilde{X}W^{\left(0\right)}\right),$$

$$H^{\left(2\right)}=SELU\left(\tilde{P}H^{\left(1\right)}W^{\left(1\right)}\right),$$

where $\tilde{A}=A+I,{\tilde{D}}_{ii}=\sum_{j}{\tilde{A}}_{ij},\tilde{P}={\tilde{D}}^{-1/2}\tilde{A}{\tilde{D}}^{-1/2}.$ is the normalized adjacency matrix, and $W^{\left(0\right)}$, $W^{\left(1\right)}$ are learnable weights. The output $H^{\left(2\right)}$ represents the final node embeddings.

The Cox proportional hazards model uses these embeddings to predict survival risk, and the network is optimized by minimizing the negative partial log-likelihood:

$$L_{Cox}=-\frac{1}{N_{E}}\sum_{i:E_{i}=1}\left(r_{i}-\log\sum_{j\in R_{i}}exp(r_{j})\right),$$

where $E_{i}$ is the event indicator, $t_{i}$ denotes the observed survival time, $N_{E=1}$ is the number of uncensored samples, $r_{i}=h_{i}^{⊤}\beta$ $R_{i}=\{j:t_{j}\geq t_{i}\}$ and β is the regression coefficient learned jointly with the GCN parameters.

### 2.2. Data and Preprocessing

We analyzed 10 TCGA cancer cohorts with matched DNA methylation, mRNA expression, and clinical survival data. The omics data were obtained as processed tabulated files rather than being generated directly from raw sequencing or array intensity files. DNA methylation data were measured using the Illumina Infinium HumanMethylation450 BeadChip platform (Illumina, Inc., San Diego, CA, USA). For each gene, the beta values ranging from 0 to 1 were averaged across its annotated CpG sites to obtain gene-level methylation features. mRNA expression data were obtained from the UNC Illumina HiSeq platform (Illumina, Inc., San Diego, CA, USA); the normalized expression values were log-transformed before model input.

To further evaluate the generalizability of the proposed model, we additionally performed external validation on an independent BRCA cohort, GSE20713_BRCA. This external cohort was used only for independent model evaluation and was not involved in feature selection, hyperparameter tuning, risk-group cutoff determination, or model training.

For missing data handling, samples without valid overall survival time or vital status were removed. Samples with more than 20% missing values in any omics modality were also excluded. For the remaining samples, missing omics values were imputed as 0 after normalization to ensure a consistent input matrix across patients and omics modalities. Only patients with matched DNA methylation, mRNA expression, and clinical survival information were retained for downstream analysis.

KEGG pathway information was obtained from KGML files downloaded from the KEGG database. For each selected KEGG pathway, the corresponding KGML file was downloaded and parsed using a custom Python (version 3.13.2) script based on the xml.etree. ElementTree module. Specifically, gene entries in the KGML files were extracted and used as graph nodes, while pathway relationships, including gene–gene or gene product–gene product interactions, were extracted and used as graph edges. In this way, each KEGG pathway was converted into a structured pathway-derived gene graph. These pathway-derived graphs were then used as prior biological structures to guide the construction of the pathway-guided gene graph in our model.

### 2.3. Baseline Methods

To evaluate the predictive performance of the proposed method, we compared it with four representative baseline methods, including traditional statistical, deep learning, and graph neural network-based survival models. All baseline methods were implemented and evaluated under the same preprocessing procedure, input features, and data partitioning strategy as the proposed model. For each TCGA cohort, the models were trained on the training/validation set, and the hyperparameters were selected according to the validation C-index during 10-fold cross-validation. The selected model was then evaluated on the independent test set.

Cox-EN: It introduces a pathwise algorithm for the Cox proportional hazards model, regularized by convex combinations of $l_{1}$ and $l_{2}$ penalties (elastic net) [22].

DeepSurv: It combines a Cox proportional hazards deep neural network with a state-of-the-art survival model to capture the interactions between a patient’s covariates and treatment effectiveness [23].

GraphSurv: It integrates a GCN with a deep Cox proportional hazards network for survival prediction [24].

LAGProg: It is a local augmented graph convolutional network that augments multi-omics data using a conditional variational autoencoder and combines it with a two-layer GCN and a Cox proportional hazard network for cancer prognosis [12].

Although both PANA-Surv and LAGProg use local neighborhood information for multi-omics survival prediction, the two methods differ in how the local neighborhood is constructed and used. LAGProg mainly performs local data augmentation based on the original neighborhood structure and then combines the augmented features with a GCN-based Cox model. In contrast, PANA-Surv explicitly reconstructs the local neighborhood of each central gene node using a top-K similarity-based selection strategy. After neighborhood reconstruction, PANA-Surv further assigns adaptive weights to selected neighboring nodes according to their similarity with the central node, so that more informative neighbors contribute more strongly to the augmented representation. Therefore, compared with LAGProg, PANA-Surv introduces a more flexible and biologically guided neighborhood reconstruction and weighting mechanism before graph convolution and survival risk prediction.

### 2.4. Cross-Validation Setup

To ensure a fair comparison and avoid information leakage, the same data partitioning strategy was applied to all methods. Each TCGA cohort was first split into an 80% training/validation set and a 20% independent test set using stratified sampling, preserving the proportion of events (status = 1) and censored observations (status = 0). All feature selection procedures, model tuning, and risk-group cutoff determination were performed only within the training/validation set. We then performed 10-fold cross-validation on the training/validation set to select the optimal model and hyperparameters according to the validation Concordance Index (C-index). The independent test set was not used during feature selection, model tuning, or cutoff determination, and was used only for final performance evaluation.

### 2.5. Performance Evaluation

The C-index evaluates the discriminative ability of survival prediction models. It measures whether patients with higher predicted risk scores tend to experience events earlier. The C-index is calculated as:

$$C-index=\frac{\sum_{i,j}1(t_{i}<t_{j})1(E_{i}=1)1(r_{i}>r_{j})}{\sum_{i,j}1(t_{i}<t_{j})1(E_{i}=1)}$$

where $t_{i}$ and $t_{j}$ denote the observed survival times of patients i and j, respectively; $E_{i}$ is the event indicator, with $E_{i}=1$ indicating that the event was observed and $E_{i}=0$ indicating censoring; and $r_{i}$ and $r_{j}$ are the predicted risk scores. A patient with a higher risk score is expected to have a shorter survival time. The denominator represents the number of comparable patient pairs. The C-index ranges from 0 to 1, with a higher value indicating better predictive performance [25].

### 2.6. Experiment Designs

#### Selection of the Neighborhood Size k

In each dataset, we examined the degree distribution of gene nodes in the KEGG pathway network to determine the appropriate range of k values. *FGF20* exhibited the highest degree with 45 connections, while *EGLN2* had the lowest degree with only one connection. Notably, no gene nodes had degrees between 27 and 45, and degrees of 45 or 1 were considered outliers. Based on this distribution, we evaluated model performance under six different k values (5, 10, 15, 20, 25, and 30) across 10 TCGA datasets.

### 2.7. Design of Ablation Variants Within PANA-VAE

We designed two ablated variants to evaluate the contribution of each mechanism within PANA-VAE.

**No-NR (no neighborhood reconstruction mechanism):** The similarity-based neighborhood reconstruction (NR) mechanism (corresponding to Figure 1f) was removed, and all neighbors defined by KEGG were used for feature augmentation. The adaptive weighting (AW) mechanism remained, and weights were computed for all neighbors.

**No-AW (no adaptive weighting mechanism):** We retained NR but removed the AW mechanism (corresponding to Figure 1g), assigning equal weights to all selected neighbors.

### 2.8. Comparison of Graph Construction Strategies

The impact of graph topology was further examined by replacing the pathway-guided graph in PANA-Surv with two alternatives: a data-driven kNN graph and a directed graph, while keeping all other components unchanged.

**KNN-based graph construction:** For each central node, the top-k most similar nodes in the feature space were selected as neighbors, and PANA-VAE performed feature augmentation on the resulting completed graph.

**Directed graph construction:** We also constructed a directed graph based on the information flow during feature augmentation. In the NR mechanism, when node *B* is selected as a neighbor of node *A* and assigned a non-zero weight by AW, a directed edge from *B* to *A* is introduced; otherwise, the edge between *B* and *A* has no direction.

For all graph construction strategies, we evaluated neighborhood sizes k∈{5,10,15,20,25,30} to examine the robustness of our method under different neighborhood sizes and to compare model performance across these settings.

### 2.9. Downstream Analyses

#### 2.9.1. Kaplan–Meier Curves

Kaplan–Meier survival curves are used to estimate the survival probabilities of different groups. The survival function is defined as $S(t)=\prod_{t_{i}\leq t}\left(1-d_{i}/n_{i}\right)$, where S(t) denotes the probability of surviving beyond time t, $d_{i}$ represents the number of events (e.g., deaths) occurring at time $t_{i}$, and $n_{i}$ is the number of individuals at risk at time $t_{i}$. The product is taken over all time points $t_{i}$ such that $t_{i}\leq t$, where $t_{i}$ are ordered in increasing time [26].

#### 2.9.2. Differential Gene Expression Analysis

We applied differential gene expression analysis to identify candidate prognostic biomarkers associated with the risk stratification produced by PANA-Surv. Specifically, PANA-Surv first generated a survival risk score for each BRCA patient. Patients were then divided into high-risk and low-risk groups according to the median predicted risk score. Differential expression analysis was performed between the high-risk and low-risk groups to identify genes with significant expression changes. The fold change (FC) represents the ratio of gene expression levels between the high-risk and low-risk groups, and the log2 fold change (log_2_FC) was calculated as follows: ${log}_{2}(FC)={log}_{2}\left(X_{\mathrm{condition}\;1}/X_{\mathrm{condition}\;2}\right),$ where $X_{\mathrm{condition}\;1}$ and $X_{\mathrm{condition}\;2}$ denote the normalized expression levels of a given gene under two different conditions. A higher absolute ${log}_{2}(FC)$ value indicates a more substantial differential expression.

R package *DESeq2* (version 1.46.0) [27] was used to compute the ${log}_{2}$(FC) and *p*-values, considering both biological and technical variability. The selection criteria for differentially expressed genes (DEGs) were adjusted *p*-values <0.05, and $|{log}_{2}(FC)|>2$.

#### 2.9.3. Enrichment Analysis

We utilized the R package *clusterProfiler* (version 4.14.6) [28] to conduct functional enrichment analysis of genes, including Gene Ontology (GO) and KEGG pathway analysis. Gene ID conversion from the HUGO Gene Nomenclature Committee (HGNC) to Ensembl and Entrez IDs was carried out using the R package *biomaRt* (version 2.62.1) [29]. Finally, we applied the Benjamini–Hochberg (BH) [30] method for multiple testing correction, considering GO terms and KEGG pathways with a q-value less than 0.05 as statistically significant.

## 3. Results

In this section, we evaluate PANA-Surv from multiple perspectives to verify its predictive performance, model design, and biological interpretability.

### 3.1. Overall Survival Prediction Performance

We compared PANA-Surv with representative survival models from three methodological categories, including the traditional statistical model Cox-EN, the deep learning model DeepSurv, and two GNN-based models, GraphSurv and LAGProg. For a fair comparison, all models used the same patient-level multi-omics features, including DNA methylation and mRNA expression profiles, together with the corresponding overall survival time and survival status for model training and evaluation. For graph-based methods, including PANA-Surv, pathway-derived graph structures were further used to model gene-level relationships. It should be noted that the output of each survival prediction model is a patient-specific risk score, which is used to evaluate patient prognosis and calculate the C-index, rather than directly predicting the exact survival time.

Table 1 summarizes the C-index values across 10 TCGA cohorts and reports the corresponding mean C-index values. PANA-Surv reached the highest mean C-index (0.6763) among all models, outperforming Cox-EN (0.5870), DeepSurv (0.5579), GraphSurv (0.5938), and LAGProg (0.6035). Compared with LAGProg, which also employs local neighborhood augmentation, PANA-Surv yielded a 12.06% improvement in mean C-index (0.6763 vs. 0.6035). Pairwise Wilcoxon signed-rank tests between PANA-Surv and the other models resulted in p-values below 0.01, indicating statistically significant differences across the 10 TCGA cohorts.

To further assess external generalizability, we evaluated the trained model on the independent GSE20713_BRCA cohort. As shown in Table 2, PANA-Surv achieved a C-index of 0.6240, which was slightly higher than GraphSurv (0.6181) and higher than CoxNN (0.5808), DeepSurv (0.5723), and LAGProg (0.4375). These results suggest that PANA-Surv maintains competitive predictive performance on an independent BRCA validation cohort.

### 3.2. Ablation Experiments Within PANA-VAE

After validating the overall performance of PANA-Surv, we conducted ablation experiments to evaluate the contribution of each mechanism within PANA-VAE.

**Figure 2:**
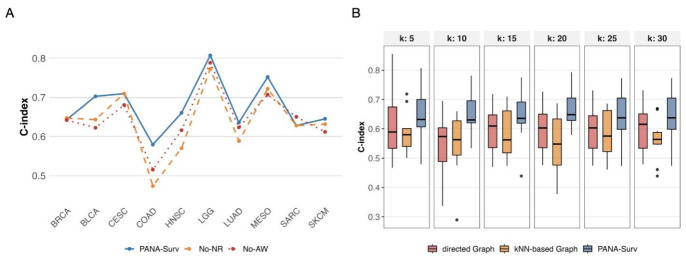
Evaluation of PANA-Surv through ablation and graph topology analyses. (**A**) C-index comparison of the full model and its two ablated variants, No-NR (without neighborhood reconstruction mechanism) and No-AW (without adaptive weighting mechanism), across 10 TCGA cancer cohorts. (**B**) Comparison of directed graph construction, kNN-based graph construction, and PANA-Surv (pathway-guided construction) under different neighborhood sizes (k).

The results are shown in Figure 2A. Both variants produced lower predictive performance than the full model. Removing neighborhood reconstruction reduced the mean C-index from 0.6763 to 0.6386, while replacing adaptive weighting decreased it to 0.6467. These results demonstrate that both mechanisms contribute to improving model performance.

### 3.3. Effectiveness of Pathway-Guided Graph Construction

We next examined how different graph construction strategies affect survival prediction performance. Based on all 10 TCGA cohorts and 6 neighborhood sizes, PANA-Surv reached the highest overall mean C-index (0.67±0.03), followed by the kNN-based (0.61±0.04) and directed graphs (0.60±0.04). As illustrated in Figure 2B, PANA-Surv maintained higher C-index values across different neighborhood sizes (k), while the two alternative graphs showed larger variation and lower stability. The kNN-based and directed graphs were more sensitive to the choice of k, suggesting that they may introduce noisy or unreliable connections.

### 3.4. Clinical Validation Through Risk Stratification and Survival Analysis

We assessed the prognostic performance of PANA-Surv through risk stratification and Kaplan–Meier survival analysis. In each cohort, patients were divided into high-risk and low-risk groups according to the median predicted risk score. Survival differences between the two groups were evaluated using the log-rank test.

**Figure 3:**
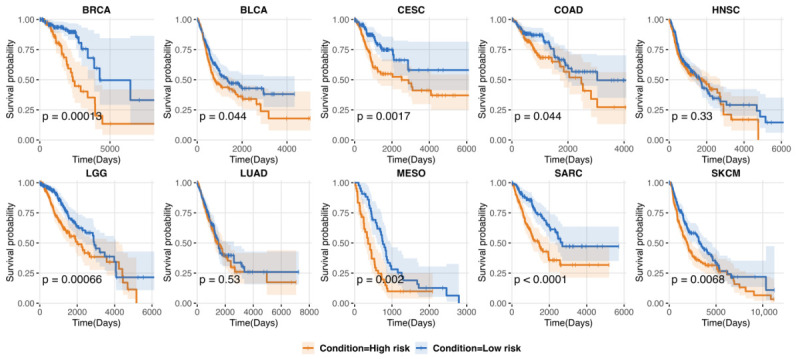
Kaplan–Meier survival curves predicted by PANA-Surv across 10 TCGA cancer cohorts. Patients were divided into high-risk (orange) and low-risk (blue) groups according to the median predicted risk score. Shaded regions represent 95% confidence intervals, and *p*-values were calculated using the log-rank test.

As shown in Figure 3, PANA-Surv successfully separated patients into groups with distinct survival outcomes in most cohorts. Significant differences were observed in BLCA (p = 0.044), BRCA (p = 0.00013), CESC (p = 0.0017), COAD (p = 0.044), LGG (p = 0.00068), MESO (p = 0.0091), SARC (p < 0.0001), and SKCM (p = 0.00068), where high-risk groups consistently exhibited shorter survival times. Although HNSC and LUAD did not reach statistical significance, their curves still demonstrated clear downward trends for high-risk patients, indicating consistent risk separation patterns.

Overall, PANA-Surv achieved consistent and reliable risk stratification across multiple cancer types, demonstrating robust performance in distinguishing patients with different survival outcomes.

### 3.5. Biological Interpretability and Downstream Analyses in BRCA

To investigate the biological interpretability of PANA-Surv, we conducted downstream analyses on the BRCA cohort.

**Figure 4:**
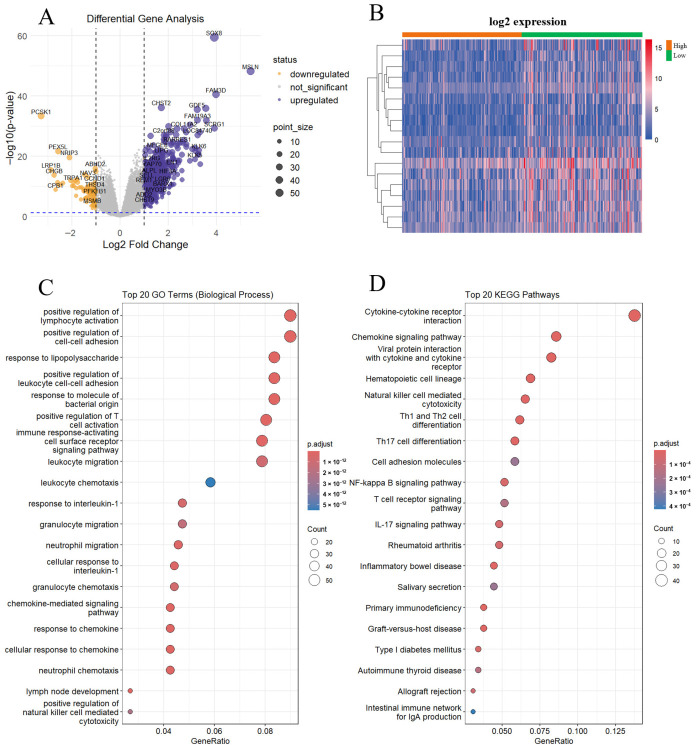
Downstream biomarker analysis and functional enrichment in BRCA. (**A**) Differentially expressed genes, where the blue dashed horizontal line represents p-value = 0.05, and the black dashed vertical lines indicate |logFC| = 2. The yellow and purple dots correspond to the significantly down-regulated and up-regulated genes, respectively. (**B**) Heatmap of identified biological features based on up-regulated and down-regulated genes filtered by PANA-Surv. (**C**) GO enrichment analysis showing the top 20 biological processes. (**D**) KEGG enrichment analysis showing the top 20 enriched pathways.

As shown in Figure 4A, differential gene expression analysis identified 723 genes ($|{log}_{2}(FC)|>2$, adjusted p<0.05), including 626 upregulated and 97 downregulated genes. The corresponding heatmap (Figure 4B) illustrates a clear separation between the two risk groups, reflecting distinct transcriptomic profiles captured by the model.

Among the 18 prognostic candidate biomarkers identified in the BRCA cohort (Table 3), 12 (66.7%) have been previously reported to be associated with breast cancer, including *MSLN*, *FAM3D*, *SOX8*, *A2ML1*, *GFRA3*, *KLK6*, *AQP5*, *FBN3*, *SLC6A14*, *KLK5*, *LRP1B* and *PCDH10*. For instance, *KLK6* has been characterized as a tumor suppressor in breast cancer [31], and elevated *SLC6A14* expression has been linked to advanced clinical stages [32]. It should be noted that Table 3 is not intended to represent a list of frequently mutated or canonical driver genes in breast cancer. Instead, the genes listed in Table 3 were selected as expression-level prognostic biomarkers based on the model-defined risk groups, differential expression analysis, statistical significance, and model ranking results. Therefore, the absence of well-known breast cancer-related genes, such as *TP53* (p53), *BRCA1/2*, *PTEN*, *ATM*, and *CHEK* family genes, does not indicate that these genes are unrelated to BRCA or deny their established roles in breast cancer. Rather, it suggests that the biomarkers identified by PANA-Surv mainly reflect risk-associated expression changes, pathway perturbations, and model-driven prognostic signals under the current multi-omics intersected gene set and KEGG pathway constraints. These findings may provide complementary information to traditional mutation-driven biomarkers.

To further evaluate the applicability of PANA-Surv to another cancer type, we performed additional biomarker analysis in the LGG cohort (Table 4). A total of 20 prognostic candidate biomarkers were identified in the LGG cohort using the same screening criteria. Among them, 15 genes, including *NECAB1*, *RASGRF2*, *CDKL2*, *RGS4*, *PAK1*, *SERPINI1*, *CAMK2A*, *SLC12A5*, *ITPR1*, *POSTN*, *H19*, *HOXC13*, *HOXA9*, *HOXC10*, and *MMP7*, have been previously implicated in glioma- or LGG-related biological processes or prognosis. The remaining five genes, *KCNJ3*, *NAPB*, *PAX3*, *TRPM8*, and *MAGEA6*, also satisfied the same screening criteria but currently lack direct glioma- or LGG-related literature support in our literature check; therefore, they are reported as potential candidate biomarkers requiring further validation. Compared with the BRCA results, the identified LGG biomarkers showed a distinct cancer-type-specific pattern, suggesting that PANA-Surv can identify biologically meaningful prognostic candidate biomarkers across different cancer contexts.

Gene Ontology (GO) enrichment analysis (Figure 4C) showed that the top biological processes were primarily related to immune activation, chemotaxis, and cell adhesion [56], such as “positive regulation of T cell activation”, “immune response-activating cell surface receptor signaling pathway” and “positive regulation of cell–cell adhesion”. Consistently, KEGG pathway enrichment (Figure 4D) revealed significant involvement of immune and inflammation-related pathways, including “T cell receptor signaling”, “Cytokine-cytokine receptor interaction”, “Natural killer cell-mediated cytotoxicity”, “NF-kappa B signaling”, and “IL-17 signaling”. These pathways highlight the roles of immune regulation and inflammatory activity in breast cancer progression [57,58,59].

These analyses demonstrate that PANA-Surv captures biologically meaningful molecular patterns underlying survival differences and highlights potential biomarkers and pathways relevant to breast cancer progression.

## 4. Discussion

This study demonstrates that integrating pathway prior knowledge with adaptive local representation learning can improve multi-omics survival prediction. PANA-Surv consistently outperformed traditional and deep learning models such as Cox-EN and DeepSurv, suggesting that pathway-guided graph modeling provides a more effective way to capture gene-level molecular dependencies associated with patient survival. Compared with existing GNN-based survival models, including GraphSurv and LAGProg, PANA-Surv further improved prognostic performance across multiple TCGA cohorts.

A key distinction between PANA-Surv and previous local augmentation methods is that many existing approaches implicitly assume that neighboring nodes contribute equally during feature generation. In contrast, PANA-Surv incorporates both neighborhood reconstruction (NR) and adaptive weighting (AW), allowing the model to retain informative neighbors while reducing the influence of weakly related or noisy local signals. The ablation experiments further support the value of this design, as removing NR or replacing AW with uniform weighting led to consistent reductions in predictive performance.

Our comparison of graph construction strategies also showed that KEGG pathway-guided graphs were more effective than both data-driven kNN graphs and directed graph variants. This result suggests that biologically curated pathway structure provides a more reliable prior for multi-omics prognostic modeling and helps reduce spurious associations that may arise in high-dimensional molecular data. In addition, PANA-Surv maintained relatively stable performance across different neighborhood sizes, indicating that the framework is reasonably robust to parameter selection.

The downstream analyses in the BRCA cohort further indicate that PANA-Surv captures biologically relevant molecular patterns associated with survival differences. The identified prognostic genes included both previously reported biomarkers and several potentially novel candidates, while the enrichment results highlighted immune- and inflammation-related pathways that are consistent with the current understanding of breast cancer progression. These findings suggest that adaptive local representation enhancement may improve not only predictive accuracy but also the biological interpretability of pathway-guided survival models.

Several limitations should also be noted. First, although KEGG pathways provide biologically meaningful prior knowledge, they do not fully capture the complexity of gene regulation and molecular interactions in cancer. Integrating additional resources, such as protein–protein interaction networks or gene regulatory networks, may further improve graph representation quality. Second, the current framework focuses on overall survival and does not explicitly model competing risks, treatment heterogeneity, or temporal disease dynamics. Extending the method to more complex clinical outcome settings may improve its applicability in future studies.

## 5. Conclusions

In summary, PANA-Surv provides a pathway-guided framework for multi-omics cancer survival analysis by integrating KEGG pathway knowledge with adaptive local feature enhancement. Through neighborhood reconstruction and adaptive weighting, the proposed method reduces noisy local propagation and improves the representation of biologically relevant neighborhood information, leading to more reliable prognostic prediction. Across 10 TCGA cancer cohorts, PANA-Surv achieved superior overall performance compared with representative traditional, deep learning, and graph-based survival models. In addition, the downstream analysis in BRCA showed that the framework can help identify prognostic genes and pathway-level biological signals associated with cancer outcomes. These results suggest that PANA-Surv offers an effective bioinformatics strategy for pathway-guided multi-omics prognostic modeling.

## Figures and Tables

**Table 1:** Summary of model performance across 10 TCGA cancer cohorts. The table reports the C-index values of PANA-Surv and four baseline methods, including Cox-EN, DeepSurv, GraphSurv, and LAGProg, grouped by model category. Bold values indicate the highest C-index among the compared methods for each cancer cohort and the highest mean C-index across all cohorts. The penultimate row reports the mean C-index across the 10 cohorts, and the last row presents the paired Wilcoxon test *p*-values comparing PANA-Surv with each baseline method; “-“ indicates that the comparison is not applicable.

	Traditional	Deep Learning	Graph Neural Networks		
Cancer Type	Cox-EN	DeepSurv	GraphSurv	LAGProg	PANA-Surv
**BRCA**	0.5145	0.6506	0.5956	0.5699	**0.6433**
**BLCA**	0.5191	0.5258	0.5630	0.5802	**0.7028**
**CESC**	0.5560	0.5192	0.5670	**0.7199**	0.7096
**COAD**	0.4780	0.5519	0.4973	0.5590	**0.5792**
**HNSC**	0.5742	0.5637	0.5620	0.6135	**0.6603**
**LGG**	0.7843	0.5885	0.7425	0.7795	**0.8071**
**LUAD**	0.5939	0.4915	0.5767	0.5581	**0.6359**
**MESO**	0.6388	0.6692	0.5940	0.5564	**0.7519**
**SARC**	0.6190	0.4888	**0.6310**	0.5176	0.6278
**SKCM**	0.5917	0.5297	0.6093	0.5805	**0.6452**
**Mean**	0.5870	0.5579	0.5938	0.6035	**0.6763**
***p*** **-value**	8.30 × 10^−4^	5.21 × 10^−4^	7.32 × 10^−4^	3.62 × 10^−3^	-

**Table 2:** External validation performance on the independent GSE20713_BRCA cohort. The table reports the C-index values of PANA-Surv and four baseline methods grouped by model category. Bold values indicate the highest C-index among the compared methods in the external validation cohort.

	Traditional	Deep Learning	Graph Neural Networks		
Cancer Type	Cox-EN	DeepSurv	GraphSurv	LAGProg	PANA-Surv
**BRCA**	0.5808	0.5723	0.6181	0.4375	**0.6240**

**Table 3:** Prognostic candidate biomarkers identified by PANA-Surv in the BRCA cohort. Eighteen prognostic candidate biomarkers were identified in the BRCA cohort based on an absolute log-fold change (|logFC|) greater than 2 and an adjusted *p*-value below 0.05. The “Reference” column indicates whether direct literature support for the association of each gene with breast cancer or BRCA-related prognosis was identified. A dash indicates that no direct BRCA-related literature support was identified in our literature check; these genes are reported as potential candidate biomarkers requiring further validation.

Gene	logFC	Adjusted *p*	Reference	Gene	logFC	Adjusted *p*	Reference
*MSLN*	5.416	5.81 × 10^−49^	[33]	*AQP5*	3.252	3.04 × 10^−21^	[34]
*FAM3D*	3.981	3.63 × 10^−41^	[35]	*FBN3*	3.135	2.12 × 10^−23^	[36]
*SOX8*	3.904	3.66 × 10^−60^	[37]	*SLC6A14*	3.116	1.87 × 10^−22^	[32]
*A2ML1*	3.317	3.56 × 10^−18^	[38]	*KLK5*	3.111	1.04 × 10^−19^	[39]
*GFRA3*	3.313	9.29 × 10^−29^	[40]	*LRP1B*	−2.872	3.54 × 10^−16^	[41]
*KLK6*	3.274	1.19 × 10^−22^	[31]	*PCDH10*	−2.072	1.13 × 10^−12^	[42]
*SCRG1*	3.930	6.13 × 10^−30^	-	*PCSK1*	−3.280	4.96 × 10^−34^	-
*CHGB*	−2.739	1.96 × 10^−14^	-	*CPB1*	−2.679	1.31 × 10^−9^	-
*SLC30A8*	−2.524	3.34 × 10^−11^	-	*CPA6*	−2.393	7.74 × 10^−12^	-

**Table 4:** Prognostic candidate biomarkers identified by PANA-Surv in the LGG cohort. Twenty prognostic candidate biomarkers were identified in the LGG cohort based on an absolute log-fold change (|logFC|) greater than 2 and an adjusted *p*-value below 0.05. The “Reference” column indicates whether direct literature support for the association of each gene with glioma or LGG-related prognosis was identified. A dash indicates that no direct glioma- or LGG-related literature support was identified in our literature check; these genes are reported as potential candidate biomarkers requiring further validation.

Gene	logFC	Adjusted *p*	Reference	Gene	logFC	Adjusted *p*	Reference
*NECAB1*	4.238	1.95 × 10^−41^	[43]	*ITPR1*	2.504	3.02 × 10^−38^	[44]
*RASGRF2*	3.269	4.64 × 10^−41^	[44]	*POSTN*	−4.015	1.48 × 10^−45^	[45]
*CDKL2*	2.937	8.96 × 10^−32^	[46]	*H19*	−3.856	6.32 × 10^−74^	[47]
*RGS4*	2.103	3.56 × 10^−18^	[48]	*HOXC13*	−3.812	5.18 × 10^−12^	[49]
*PAK1*	2.480	6.32 × 10^−36^	[50]	*HOXA9*	−2.368	2.87 × 10^−11^	[51]
*SERPINI1*	2.497	2.16 × 10^−37^	[52]	*HOXC10*	−2.296	7.41 × 10^−8^	[53]
*CAMK2A*	2.303	2.02 × 10^−34^	[52]	*MMP7*	−2.291	3.67 × 10^−20^	[54]
*SLC12A5*	2.158	9.40 × 10^−33^	[55]	*PAX3*	−3.801	2.20 × 10^−14^	-
*KCNJ3*	3.222	7.59 × 10^−41^	-	*TRPM8*	−2.381	5.79 × 10^−23^	-
*NAPB*	2.393	1.37 × 10^−35^	-	*MAGEA6*	−2.251	1.92 × 10^−10^	-

## Data Availability

The data presented in this study were derived from public domain resources. TCGA multi-omics and clinical data are available from the Genomic Data Commons (GDC) portal, and KEGG pathway data were obtained from the KEGG database. The code used in this study is publicly available at https://github.com/hebut-xiaowencao/PANA-Surv_cancer_prognosis, accessed on 16 April 2026.

## Abbreviations

AW	Adaptive weighting
BH	Benjamini–Hochberg
BLCA	Bladder urothelial carcinoma
BP	Biological process
BRCA	Breast invasive carcinoma
CESC	Cervical squamous cell carcinoma and endocervical adenocarcinoma
C-index	Concordance index
COAD	Colon adenocarcinoma
DEG	Differentially expressed gene(s)
ELBO	Evidence lower bound
EN	Elastic net
FC	Fold change
GNN	Graph neural network
GO	Gene ontology
HGNC	HUGO Gene Nomenclature Committee
HNSC	Head and neck squamous cell carcinoma
KEGG	Kyoto Encyclopedia of Genes and Genomes
KGML	KEGG markup language
KNN	k-nearest neighbors
LAGProg	Local augmented graph for cancer prognosis
LGG	Brain lower grade glioma
LUAD	Lung adenocarcinoma
MESO	Mesothelioma
NR	Neighborhood reconstruction
PANA	Pathway-guided adaptive neighborhood augmentation
PPI	Protein–protein interaction
SARC	Sarcoma
SKCM	Skin cutaneous melanoma
TCGA	The Cancer Genome Atlas
UNC	University of North Carolina
VAE	Variational auto-encoder
*MSLN*	Mesothelin
*FAM3D*	Family with sequence similarity 3 member D
*SOX8*	SRY-box transcription factor 8
*A2ML1*	Alpha-2-macroglobulin like 1
*SLC30A8*	Solute carrier family 30 member 8
*PCSK1*	Proprotein convertase subtilisin/kexin type 1
*CPB1*	Carboxypeptidase B1
*CPA6*	Carboxypeptidase A6
*NECAB1*	N-terminal EF-hand calcium binding protein 1
*RASGRF2*	Ras protein specific guanine nucleotide releasing factor 2
*CDKL2*	Cyclin dependent kinase like 2
*RGS4*	Regulator of G protein signaling 4
*PAK1*	p21 (RAC1) activated kinase 1
*SERPINI1*	Serpin family I member 1
*CAMK2A*	Calcium/calmodulin dependent protein kinase II alpha
*SLC12A5*	Solute carrier family 12 member 5
*ITPR1*	Inositol 1,4,5-trisphosphate receptor type 1
*POSTN*	Periostin
*H19*	H19 imprinted maternally expressed transcript
*HOXC13*	Homeobox C13
*HOXA9*	Homeobox A9
*HOXC10*	Homeobox C10
*MMP7*	Matrix metallopeptidase 7
*KCNJ3*	Potassium inwardly rectifying channel subfamily J member 3
*NAPB*	NSF attachment protein beta
*TRPM8*	Transient receptor potential cation channel subfamily M member 8
